# Recent Progress Towards a Gonococcal Vaccine

**DOI:** 10.3389/fcimb.2022.881392

**Published:** 2022-04-11

**Authors:** Stavros A. Maurakis, Cynthia Nau Cornelissen

**Affiliations:** Institute for Biomedical Sciences, Georgia State University, Atlanta, GA, United States

**Keywords:** *Neisseria gonorrhoeae*, gonorrhea, vaccine, sexually-transmitted infections, immunity, epitope, computation, modeling

## Abstract

Gonorrhea is a global health concern. Its etiological agent, *Neisseria gonorrhoeae*, rapidly acquires antimicrobial resistance and does not confer protective immunity as a consequence of infection. Attempts to generate an effective vaccine for gonorrhea have thus far been unsuccessful, as many structures on the bacterial envelope have the propensity to rapidly change, thus complicating recognition by the human immune system. In response to recent efforts from global health authorities to spur the efforts towards development of a vaccine, several new and promising steps have been made towards this goal, aided by advancements in computational epitope identification and prediction methods. Here, we provide a short review of recent progress towards a viable gonococcal vaccine, with a focus on antigen identification and characterization, and discuss a few of the tools that may be important in furthering these efforts.

## Introduction

*Neisseria gonorrhoeae* (Ngo) causes the common sexually-transmitted infection (STI) gonorrhea. Since its first isolation in the late nineteenth century, Ngo has become an increasingly urgent global health threat. Ngo afflicted an estimated 87 million people in 2016 (Rowley et al., 2019), and the organism has to date developed resistance to every class of antimicrobial drug used for treatment (Unemo et al., 2019). Gonorrhea infects both men and women. In men, complications can include prostatitis, urethritis, and epididymitis, and in women pelvic inflammatory disease, infertility, or ectopic pregnancy. Infection by, and subsequent clearance of Ngo does not confer a protective immune response (Liu et al., 2011), so reinfection is common. Progress towards an effective gonococcal vaccine has been slow, as the propensity of Ngo for high-frequency phase and antigenic variation makes identification of suitable immunogens difficult (Criss et al., 2005; Zhu et al., 2011; Jerse et al., 2014). Further complicating matters is the fact that correlates of protection for gonorrhea are unknown (Russell et al., 1999). In response to these difficulties, global health authorities have redoubled efforts towards the development of an effective vaccine for gonorrhea prevention. This limited review will discuss early trials of gonococcal vaccines, recent breakthroughs in characterization of novel vaccine targets, and the emergence of bioinformatic tools which aid in this venture. In addition, it will briefly cover studies that have investigated cross protection against gonorrhea generated by meningococcal vaccines. For a more comprehensive review of gonorrhea vaccine development, including epidemiological and vaccine impact information, host-pathogen interactions, animal models, and considerations for other STI pathogens, the reader is directed towards recent reviews by Broutet et al. (2014), Russell et al. (2019) and Gottlieb et al. (2020).

## Previous Vaccine Attempts

Previous attempts to generate an effective gonococcal vaccine have been unsuccessful. As mentioned, the propensity for Ngo to alter the antigenic identity and expression levels of surface structures has made identification of vaccine targets challenging. To date only two candidate gonococcal vaccines have reached human trials. One candidate targeted pilin, the major component of the type IV pilus (Boslego et al., 1991), though no difference in protection was seen between placebo and vaccinated groups. It is likely that antigenic variability of pilin contributed to protection failures in this trial (Tramont and Boslego, 1985). The other trial utilized heat-killed, partially lysed whole gonococcal cells that, while able to generate bactericidal antibodies in a high percentage of recipients, did not show long-term protection; within one year post-vaccination, similar numbers of subjects were infected with Ngo regardless of their vaccination status (Greenberg et al., 1974; Greenberg, 1975). Despite these failures, other immunization efforts have shown that it is at least possible to reduce susceptibility to gonococcal infection in chimpanzees (Arko et al., 1976). This study suggested that natural infection generates some level of acquired immunity in cases of gonococcal urethritis or pharyngitis, as previously colonized chimps required a significantly higher bacterial dose to achieve reinfection. However, protection waned over time, and immunity was not seen when previously “immune” chimps were reinfected years later. Finally, the outer-membrane porin (PorB) has received considerable attention as a vaccine antigen due to its roles in adherence and dissemination. For example, Zhu et al. formulated with PorB as a DNA-based vaccine (Zhu et al., 2004), which expressed *porB* from plasmid, and as recombinant protein alone or presented in viral replicon particles (Zhu et al., 2005). The DNA-based vaccine was able to induce both a Th1 and Th2 response, depending on method of delivery, and the viral replicon particle-based method showed some protective capability in mice. Prior to this, an animal trial was conducted comparing porin formulated in liposomes, proteosomes, and gonococcal membrane blebs (Wetzler et al., 1992). This study found that porin delivered in liposomes, which mimics the *in vivo* structure, generated greater numbers of anti-porin antibodies, including those that recognized surface-exposed sections of porin, compared to other delivery methods. However, this study did not proceed to clinical trials. Nevertheless, progress towards a gonococcal vaccine continues, and new, promising targets for vaccination are being identified.

## TonB-Dependent Transporters

Ngo produces eight TonB-dependent transporters (TdTs), which are integral outer-membrane β-barrel transporters. Of these, three are aided by an associated lipid-modified protein tethered to the outer membrane. The TdTs play a critical role in allowing Ngo to survive in the nutrient-depleted environment encountered within the human host by allowing the pathogen access to transition metals such as iron and zinc. Upon binding their host ligands, which include iron-sequestering proteins like transferrin and lactoferrin, and zinc-sequestering proteins such as S100 proteins, the TdTs liberate their bound metal ions. The metals are then transported through the TdT barrel into the periplasm - aided by the proton motive force - and are intercepted by a cognate ABC transporter that translocates the metal into the cytoplasm (Cornelissen and Hollander, 2011; Cornelissen, 2018). Critically, the TdTs are well-conserved across Ngo and *Neisseria meningitidis* (Nme) isolates, and only a few are capable of phase variation, making them promising targets for vaccination efforts. The TdTs are in many cases capable of eliciting an antibody response (Price et al., 2004; Stork et al., 2010), and they play critical roles in Ngo survival and infection (Cornelissen et al., 1998; Hagen and Cornelissen, 2006; Calmettes et al., 2015; Jean et al., 2016). Despite their promise, it is feasible that the binding of host ligands during metal piracy may generate a quasi-mimicry of self-antigens, thus contributing to immune evasion. Interestingly, however, Frandoloso et al. showed that a nonbinding mutant of the surface lipoprotein TbpB in *Haemophilus parasuis*, which cannot interact with transferrin but still retains the overall structure of the wildtype, can generate an enhanced protective response compared to native TbpB in a porcine challenge model (Frandoloso et al., 2015). While it is unclear whether this phenomenon is transferable to the transporters themselves, Cash et al. found that mutagenesis of the gonococcal TdT TbpA could abrogate human transferrin binding without appreciable disruption of the overall TbpA structure (Cash et al., 2015). As generation of a nonbinding Ngo TbpA appears possible, using it as an immunogen to test protection relative to the wildtype may be a promising way forward. Indeed, as researchers gain more insight into other TdT/host protein pairs (Jean et al., 2016; Maurakis et al., 2019) and further characterize TdT structures and binding propensities (Sikora et al., 2018; Yadav et al., 2019; Kammerman et al., 2020), similar studies with the other TdTs are feasible.

Despite these advantages, however, large-scale production of integral transmembrane proteins such as the TdTs presents a few technical limitations that may impact commercial vaccine endeavors. 1) Such proteins are produced at relatively low levels in membranes, which necessarily slows production times. 2) Stabilization and solubilization of these proteins requires highly-optimized detergent concentrations. 3) Purification directly from membranes requires specific expertise and equipment that is not necessary for purification from the cytoplasm. To address these issues, Fegan et al. (2019) utilized an innovative approach to generate a TbpA/B hybrid antigen. This novel antigen utilizes the C-lobe of the immunogenic lipoprotein TbpB as a soluble protein “scaffold” onto which putatively immunogenic extracellular loop sections of TbpA are transplanted in place of TbpB loops. Antisera raised against these hybrid antigens recognized surface-exposed TbpA of Nme and was capable of inhibiting transferrin utilization by gonococci. Previously, Price et al. generated a similar hybrid by fusing Ngo TbpA loop 2 to the N-terminal lobe of TbpB, using the A2 domain of cholera toxin as a hosting molecule. These chimeras generated bactericidal and growth-inhibitory antibodies against Ngo (Price et al., 2007). Taken together, these studies suggest hybrid antigens as a promising solution to the technical limitations imposed by the TdTs.

## Lipooligosaccharide (LOS)

Gonococcal LOS has garnered attention recently as a feasible vaccine candidate [fully reviewed in (Gulati et al., 2019b)]. While LOS is subject to phase variation (Apicella et al., 1987), it is easily accessible on the gonococcal surface, where it is present in abundance. Furthermore, while many LOS antigens share structures with human glycosphingolipids thus making them ineligible for targeting, two distinct LOS epitopes, recognized by mAb L8 and mAb 2C7 (Gulati et al., 1996; Yamasaki et al., 1999), do not suffer this pitfall. A recent study (Ram et al., 2018) showed that the 2C7 epitope, which is widely conserved amongst gonococci, is immunogenic during natural infection. As such, the 2C7 epitope has attracted attention as a vaccine target. Due to the complications of purifying or directly synthesizing oligosaccharide structures, along with their generally poor immunogenicity, an attractive prospect for utilizing such antigens is to create a peptide mimic – a so-called “mimitope” – with a highly similar structure to the natural oligosaccharide. For 2C7, this was successfully done in 2006 (Ngampasutadol et al., 2006), and continuing work showed that immunization with this mimitope alongside a T_H_1-stimulating adjuvant resulted in generation of bactericidal IgG, reduced gonococcal colonization, and sped bacterial clearance in experimentally infected mice (Gulati et al., 2019a). As LOS plays a key role in Ngo’s pathogenic lifestyle and a 2C7-based vaccine would ostensibly offer broad cross-reactivity against gonococcal strains, this represents an important step forward in the quest for gonococcal prevention.

## Meningococcal Vaccines and OMVs

Vaccines against Nme serogroup B have been suggested to confer protection against gonococcal infections in addition to their intended meningococcal role. Various observational studies noted a decrease in gonococcal infections in regions after administration of meningococcal group B outer-membrane vesicle (OMV) vaccines (Whelan et al., 2016; Longtin et al., 2017; Ochoa-Azze, 2018), perhaps owing to the high level of genetic similarity between Ngo and Nme. In a case-control study of the Nme group B OMV vaccine administered in New Zealand, MeNZB (Arnold et al., 2011), researchers estimated the vaccine to be approximately 31% effective for gonococcal cross-protection, with effectiveness waning over time (Petousis-Harris et al., 2017). Bioinformatic analyses have compared MeNZB, which is no longer on the market, to the currently-available Nme group B vaccine Bexsero^®^ (GSK), which contains the MeNZB OMV antigen alongside *Neisseria* adhesin A (NadA), factor H binding protein (fHbp), and *Neisseria* heparin binding antigen (NHBA) (Toneatto et al., 2017). These antigens were also compared to their homologous gonococcal proteins, and assessed for their ability to generate anti-gonococcal antibodies (Semchenko et al., 2019). These analyses found that Ngo strain FA1090 encodes homologues to 20 of 22 core proteins from the Bexsero OMV, of which 16 share >90% identity with the vaccine antigen. While the *nadA* gene is absent from Ngo and fHbp is not believed to surface exposed in Ngo, NHBA from FA1090 is approximately 69% identical to the Bexsero variant. Furthermore, this study showed that OMV-derived antibodies can recognize gonococcal proteins, and others demonstrated that immunization with Bexsero^®^ accelerates Ngo clearance in a mouse challenge model (Leduc et al., 2020).

OMVs are bi-layered membrane spheres that are naturally released from Gram-negative bacteria, including Ngo, and have received considerable attention as vaccine antigens for STI pathogens (Chbib et al., 2021). Their surface is decorated with phospholipids, various outer-membrane proteins, and lipopolysaccharide/lipooligosaccharide (Kulp and Kuehn, 2010). Many common OMV proteins in addition to those discussed above share similarity between Ngo and Nme (Marjuki et al., 2019), making OMVs a promising platform for a universal pathogenic *Neisseria* vaccine. For Ngo, an OMV preparation with microencapsulated IL-12 as an adjuvant was recently shown to accelerate gonococcal clearance and to induce Ngo-specific antibodies when administered intravaginally to BALB/c mice (Liu et al., 2017). Critically, this combination conferred protection against heterologous Ngo strains (Liu et al., 2018), which is necessary for effective vaccination. In another study, an OMV from Nme that was modified to overexpress a mutant fHbp and an attenuated endotoxin was able to elicit human complement-mediated serum bactericidal antibodies against Ngo (Beernink et al., 2019). More recently, detoxified Nme OMVs as immunogens demonstrated a protective effect against gonococcal challenge in mice, which was associated with anti-OMV immunoglobulins that cross-reacted with Ngo (Matthias et al., 2021).

## NHBA and Bacterial Ghosts

The previous section briefly discussed NHBA as a component of the Nme Bexsero^®^ vaccine, but this antigen has also independently received attention as a contributor to protection against gonococci. Ngo NHBA is surface exposed and is involved in interactions with host cells (Semchenko et al., 2019). Furthermore, this antigen is conserved across Ngo genomes and has a high sequence identity among isolates. It was recently shown that recombinant NHBA is immunogenic and that anti-NHBA antibodies recognize a wide range of Ngo NHBA variants (Semchenko et al., 2020a). In addition, these antibodies promote C3 deposition and opsonophagocytosis. The biological importance of NHBA should not be overlooked, as it plays a role in formation of gonococcal microcolonies and adherence to epithelial cells (Semchenko et al., 2020b).

An interesting adjunct to vaccination platforms is that of the bacterial ghost. Ghosts are empty shells of Gram-negative bacteria produced from the expression of bacteriophage PhiX174 lysis gene E (Langemann et al., 2010). Expression of gene E in non-host-range bacteria for PhiX174 leads to conversion of Gram-negative bacteria into ghosts, rather than causing lysis. Critically, these ghosts expel their nucleic acids, cytoplasmic proteins, and ribosomes during formation, leaving behind a fused outer and inner membrane with an antigenic profile identical to that of the counterpart bacteria. As a result, these ghosts can be utilized for immunization efforts involving envelope antigens, and can even be adapted as delivery vehicles for molecules such as soluble protein or DNA (Muhammad et al., 2012). Jiao et al. (2018) recently generated a *Salmonella enteriditis* ghost for the delivery of a PorB-based Ngo DNA vaccine. In this study, the eukaryotic expression plasmid pVAX1 containing a full-length *porB* gene was loaded into ghosts and used to transfect mouse bone marrow-derived dendritic cells (BMDCs), which were activated in response to the ghost. In addition, mice produced PorB-specific antibodies upon immunization, and mice immunized with the PorB ghost showed elevated CD4^+^ and CD8^+^ T-cell counts compared to those immunized with ghost alone, and both groups were higher than the control group. In a later study (Jiao et al., 2020), this *S. enteriditis* ghost was again utilized for the delivery of an Ngo *nspA*-based vaccine. The *nspA* gene was cloned into plasmid and loaded into the ghost, which was used to immunize BALB/c mice either alone, or alongside ghosts containing the Ngo *porB* gene, as previous reports showed PorB to be an effective adjuvant for this system when delivering *S. enteriditis* antigens (Jiao et al., 2016). When co-administered, these ghosts elicited specific IgG and stimulated lymphocyte proliferation. Therefore, the bacterial ghost delivery method may be a promising approach for future Ngo vaccine studies.

## Epitope Prediction Tools

**Figure 1:**
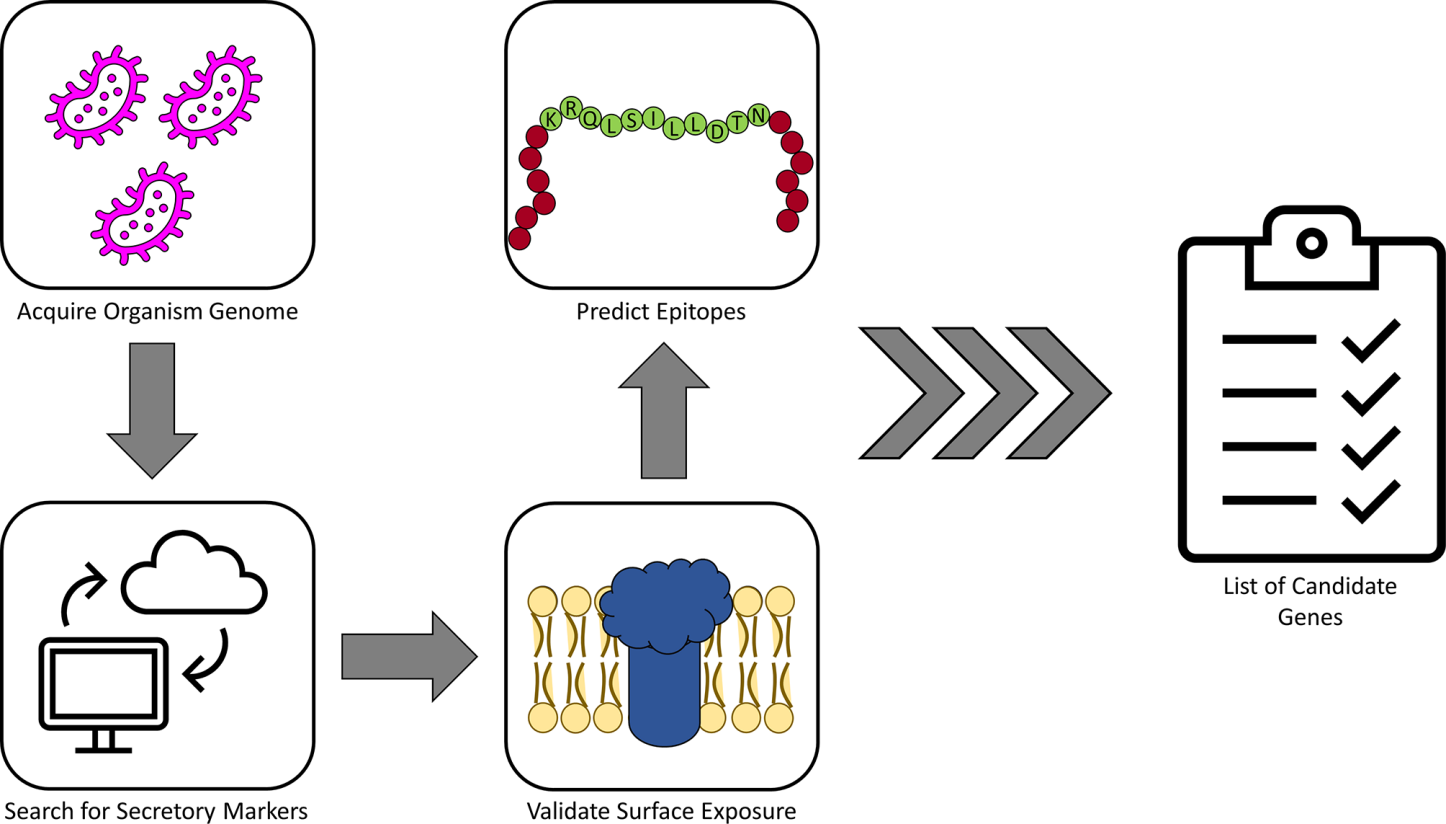
A simplified pipeline for identifying candidate antigens. The cartoon above demonstrates a simplified version of the antigen identification pipeline described in the text. In short, once a genome has been chosen for the organism in question, this genome can be screened for genes likely to produce surface-exposed products, which represent a logical first choice in identifying targets. Once a list of potential surface targets has been identified, these can be further validated by running through topology prediction tools or similar methods to verify the initial screen. From here, the list can be further narrowed down to include only gene products with sufficient epitope characteristics, such as those for B cells and/or T cells, leaving the researcher with a finalized list of candidate targets for evaluation.

Recently, high-throughput computational methods have been utilized to speed the process of epitope prediction and characterization for vaccine development. These techniques have even been used to design synthetic particles that themselves may act as vaccine antigens. While examples of these tools are too numerous for the scope of this review, we highlight a few in this section. For example, Jain et al. (2016) utilized these methods to establish a functional pipeline for identifying possible Ngo epitopes. In short, the SignalP server (DTU Health Tech), which utilizes artificial networks and known organism proteomic databases to predict the presence and location of signal sequences and cleavage sites, was used to predict secretory proteins from Ngo. Candidates from this screen were then submitted to HMMTOP (Tusnády and Simon, 2001), a tool for membrane topology prediction, for validation. Finally, candidate proteins were analyzed by VaxiJen 2.0 for antigen prediction (Doytchinova and Flower, 2007) and by HLAPred for identification of T- and B-cell epitopes. This pipeline resulted in identification of 23 potential Ngo vaccine candidates for utilization in further characterizations. Such a pipeline may be applied to other pathogens as well; a simplified schematic of such a workflow is illustrated in **Figure 1**.

PubMLST contains a large database of Ngo genomic data. Baarda et al. (2018) outlined a methodology for “mining” Ngo alleles from PubMLST which may be applied to eventual vaccine design. In this method, one can query a given gonococcal gene and find all alleles of the gene, including their translated protein sequence, within the database. From this point, one can see sequence conservation and distribution within the population, and then proceed to species-specific polymorphism analysis. At this stage, the user can map polymorphisms onto confirmed protein structures *via* 3D protein analysis tools like PyMOL or UCSF Chimera and identify the conservation of specific antigenic targets, if any are known. In a test example of the workflow, Baarda et al. examined 3,908 Ngo genomes to identify polymorphisms in the MtrE protein. Cumulatively they found that five variants of MtrE represent 98% of MtrE diversity in Ngo, with a large percentage of the protein being conserved across all isolates. Moreover, structural mapping for MtrE revealed the locations of low and high-prevalence polymorphisms within the protein. Such analyses for MtrE and other Ngo proteins are a useful step forward in targeted, rational vaccine design based on exposed, conserved epitopes.

MtrE has been included in other computational vaccine studies as well, such as that performed by Wang et al. (2017), which utilized ferritin nanoparticles as a scaffold for antigenic MtrE loops. The self-assembling ferritin can house relatively large protein domains on its N-terminus (Kanekiyo et al., 2013), so Wang et al. used ferritin from *Helicobacter pylori* to present MtrE surface-exposed loops as vaccine antigens. These efforts showed that the chimeric molecule could assemble and present loops in a way that should allow antibody access. During the design phase of these chimeras, structural data from the MtrE crystal’s antigenic loops was copied and inserted into a model of the ferritin cage, then the preliminary chimera models were refined for thermodynamic favorability in 3Drefine (Bhattacharya et al., 2016). These chimeras were then purified, and their crystal structures were solved, showing that insertion of MtrE loop peptides did not disrupt the overall structure of the ferritin cage, suggesting that this nanoparticle method could be a suitable platform for delivering Ngo immunogens alone or in an ordered array.

## Concluding Remarks

*Neisseria gonorrhoeae* has presented challenges to researchers for decades. This pathogen causes illness in millions of humans and utilizes an arsenal of antimicrobial resistance strategies and highly-variable surface structures to make treatment and prevention an enormous challenge. Despite these difficulties, the studies described above represent promising steps forward in the effort to combat this urgent threat pathogen. Bioinformatic tools and computational methods now make antigen identification and characterization easier than ever, so it is possible that new targets will emerge as well. This may prove critical to our efforts in combating a pathogen with super bug potential.

## Author Contributions

SM performed initial literature review and manuscript drafting, editing. CC performed proofreading and funding acquisition. All authors contributed to the article and approved the submitted version.

## Funding

This work was supported by NIH grants R01 AI125421, R01 AI127793, and U19 AI144182 (all to CC). 

## Conflict of Interest

The authors declare that the research was conducted in the absence of any commercial or financial relationships that could be construed as a potential conflict of interest.

## Publisher’s Note

All claims expressed in this article are solely those of the authors and do not necessarily represent those of their affiliated organizations, or those of the publisher, the editors and the reviewers. Any product that may be evaluated in this article, or claim that may be made by its manufacturer, is not guaranteed or endorsed by the publisher.
